# Outstanding nonlinear optical properties of all-inorganic perovskite CsPbX_3_ (X=Cl, Br, I) precursor solutions and polycrystalline films

**DOI:** 10.1016/j.isci.2023.108514

**Published:** 2023-11-23

**Authors:** Yue Fu, Srinivasa Rao Konda, Rashid A. Ganeev, Vyacheslav V. Kim, Ganjaboy S. Boltaev, Ran Wang, Weili Yu, Wei Li

**Affiliations:** 1 The GPL Photonics Laboratory, State Key Laboratory of Luminescence and Applications, Changchun Institute of Optics, Fine Mechanics and Physics, Chinese Academy of Sciences, Changchun, Jilin 130033, China; 2 Microelectronics Instruments and Equipments R&D Center, Institute of Microelectronics of Chinese Academy of Sciences, Beijing 100029, China; 3 Institute of Fundamental and Applied Research, TIIAME National Research University, Kori Niyoziy 39, Tashkent 100000, Uzbekistan; 4 Laboratory of Nonlinear Optics, Institute of Astronomy, University of Latvia, Jelgavas iela 3, 1004 Riga, Latvia; 5 Department of Physics, Voronezh State University, Voronezh 394006, Russia; 6 Chirchik State Pedagogical University, 104 Amir Temur, Chirchik 111700, Uzbekistan

**Keywords:** Materials science, Optics, Physics

## Abstract

In this work, we systematically explore the third-order nonlinear optical properties of all-inorganic CsPbX_3_ (X = Cl, Br, I) perovskite precursor solutions and thin films using femtosecond and nanosecond laser pulses. We show that these samples possess strong two-photon absorption (TPA) and reverse saturable absorption (RSA), which depend on the excitation wavelength. The obtained nonlinear absorption and refraction coefficients for precursor solutions are followed by the relation CsPbCl_3_ > CsPbBr_3_ > CsPbI_3_ for the 800 and 1,064 nm excitation wavelengths, whereas this relation became reverse in the case of 355 and 400 nm laser pulses. It was shown that CsPbCl_3_ thin film possesses RSA at 400 nm, CsPbCI_3_ shows RSA+ saturable absorption (SA), and CsPbBr_3_ demonstrates SA + RSA. In addition, at 800 nm excitation, the CsPbBr_3_ thin films show SA + RSA at low peak intensity, and the absorption becomes reverse (TPA+SA) with a further increase in the input laser intensity. The suitability of nonlinear absorption depends on the band gap of the thin films with respect to the pumping photon energy. Our studies demonstrate that these perovskites can be used as the excellent materials for the all-optical signal processing.

## Highlights

• The third-order NLO properties of CsPbX_3_ (X = Cl, Br, I) perovskite were explored
• ns and fs Z-scan technique was applied to investigate the third-order NLO properties
• The effect of wavelength and composition on the NLO properties was elucidated
• RSA and TPA play pivotal roles in the NLO properties in thin film

## Graphical abstract

**Figure:**
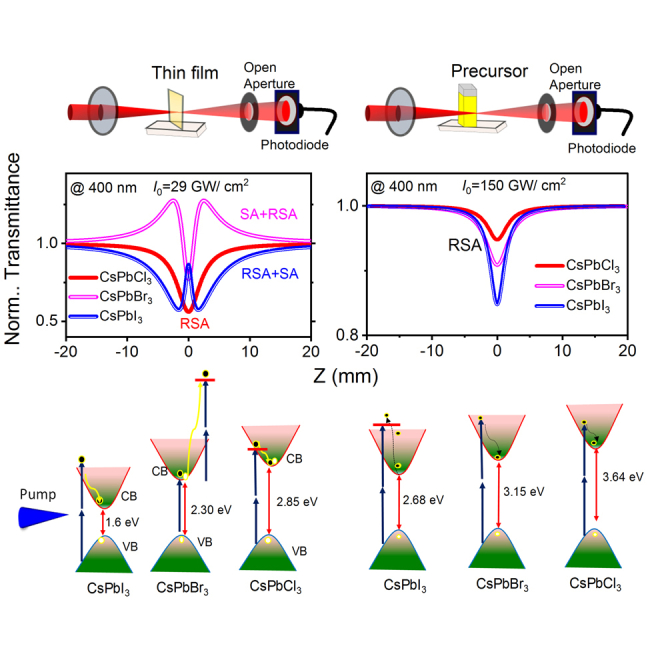

## Introduction

Halide perovskites (AMX_3_, X = Cl, Br, I) have attracted interest due to their applications in photovoltaic devices, photodetectors, light-emitting diodes (LEDs), and lasers.^1^^,^^2^^,^^3^^,^^4^^,^^5^^,^^6^^,^^7^^,^^8^^,^^9^^,^^10^ Meanwhile, the typical hybrid organic perovskites are sensitive to the outdoor applications due to their poor stability in the presence of water and oxygen. Therefore, substituting the organic cations with stable inorganic Cs^+^ ions can benefit practical applications. CsPbX_3_ perovskites have attracted enormous attention in recent years due to their excellent optoelectronic properties, outstanding thermal/light stability, performance of large carrier mobility, long charge carrier lifetime, high luminescence, diminished deep-level defects, and wide range of applications in electronic devices.^11^^,^^12^^,^^13^ The low solubility of the CsX in organic solvent prevents the formation of high-quality thin films, which impulse for their applications in various fields.^14^^,^^15^^,^^16^^,^^17^

A variety of nonlinear optical (NLO) materials, like inorganic crystals and small-sized nanoparticles, have been intensively studied and applied in the field of optical limiting,^18^ nonlinear optics,^19^^,^^20^^,^^21^^,^^22^ tunable lasing,^23^ photo refractivity applications, optical storage,^24^^,^^25^^,^^26^ etc. Halide perovskite materials have been explored as promising NLO materials featuring versatile chemical structures and large NLO coefficients.^27^^,^^28^ Recently, organometallic halide (CH_3_NH_3_PbX_3_, X = Cl, Br, I) perovskite nanocrystal films have demonstrated strong third-order NLO properties (nonlinear absorption coefficient *β* = 10^−7^ cm W^−1^, nonlinear refractive index *γ* = 10^−13^ cm^2^ W^−1^) in the mid-infrared (MIR) spectral region.^29^ The variation of the nonlinearities of halide perovskites was attributed to the different chemical and physical properties of species. The films of CsPbBr_3_ perovskite nanocubes and nanorods have shown large two-photon absorption (TPA) cross sections at 600, 700, and 800 nm,^30^ which were two orders of magnitude larger compared with the colloidal quantum dots.^31^

CsPbBr_3_ colloidal nanocrystals observed strong TPA from the open-aperture Z-scan curves. CsPbCl_3_ and CsPb (Cl_0.53_Br_0.47_)_3_ were also synthesized and compared regarding nonlinear optics by performing Z-scan measurements (from 620 to 720 nm).^32^^,^^33^^,^^34^ The Cs_4_PbBr_6_ 0-D nanocrystals achieved strong multi-photon absorption properties in the broadband region using the Z-scan techniques.^35^ However, the all-inorganic perovskite thin films are highly reported in the optoelectronic properties but without the NLO properties.

The inorganic perovskite solutions and polycrystalline films also attracted large interest due to the easiness of preparation and processing. It is critical to make the complete surface coverage of perovskite films to avoid short circuits for the better performance of CsPbX_3_-based devices. The effect of morphology and halogen on the performance of inorganic cesium lead halide perovskite-based devices is also important for the NLO studies. However, NLO properties of all-inorganic perovskites are yet to be determined in detail. In this paper, we analyze systematically the NLO properties of ternary all-inorganic cesium lead halide perovskites (CsPbX_3_) precursor solutions and thin films of perovskites using the femtosecond and nanosecond pulses.

The combination of nonlinear absorption and nonlinear refraction properties can improve the performance of photonic devices. It is more important to study NLO with different incident conditions. Although the superior NLO properties of perovskites show a wide range of application prospects, the third-order NLO properties and optical limiting properties of all-organic halide perovskite still need further exploration. The importance of this study is revealed under such excitation of different wavelengths and pulse durations and revealed the switchability of nonlinear absorption and refraction process. This further leads to exploring these materials in applications of photonic devices and optical limiters.

## Results and discussion

**Figure 1:**
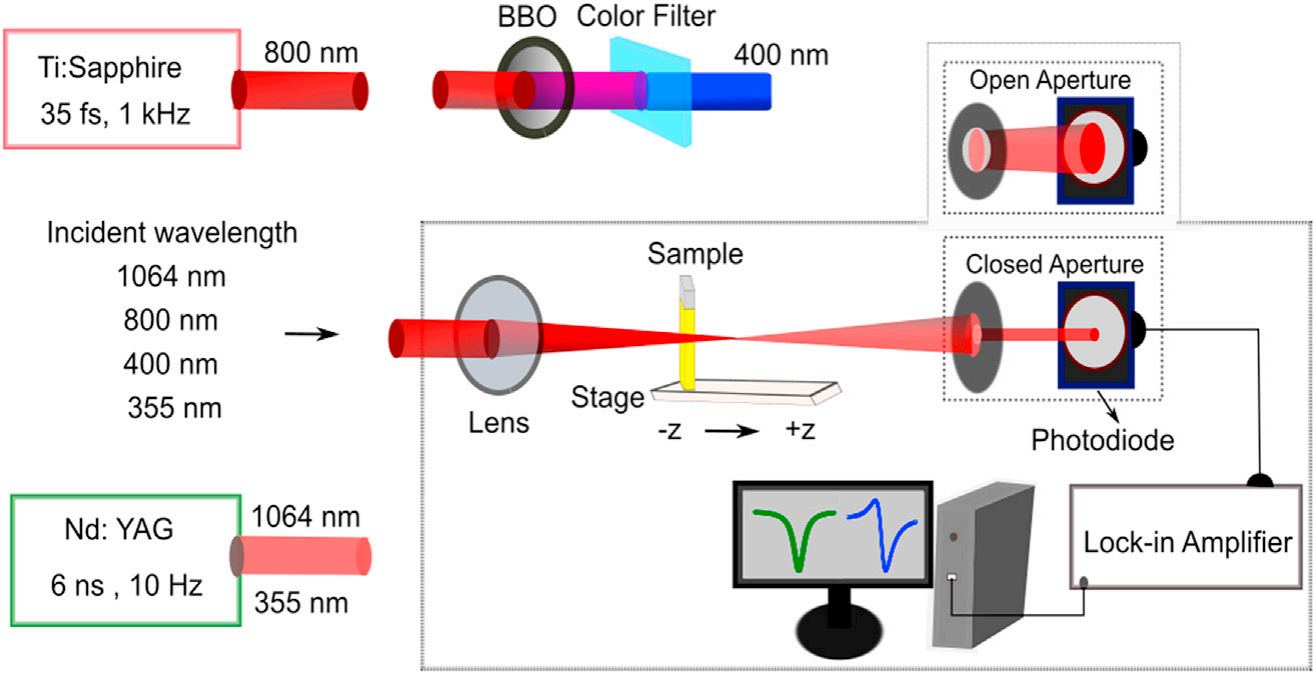
Experimental setup for Z-scan measurements

The experimental setup used for Z-scan measurements is shown in Figure 1. The complete description is presented in the experimental model and study participant details.

### Morphological and optical characterization of samples

**Figure 2:**
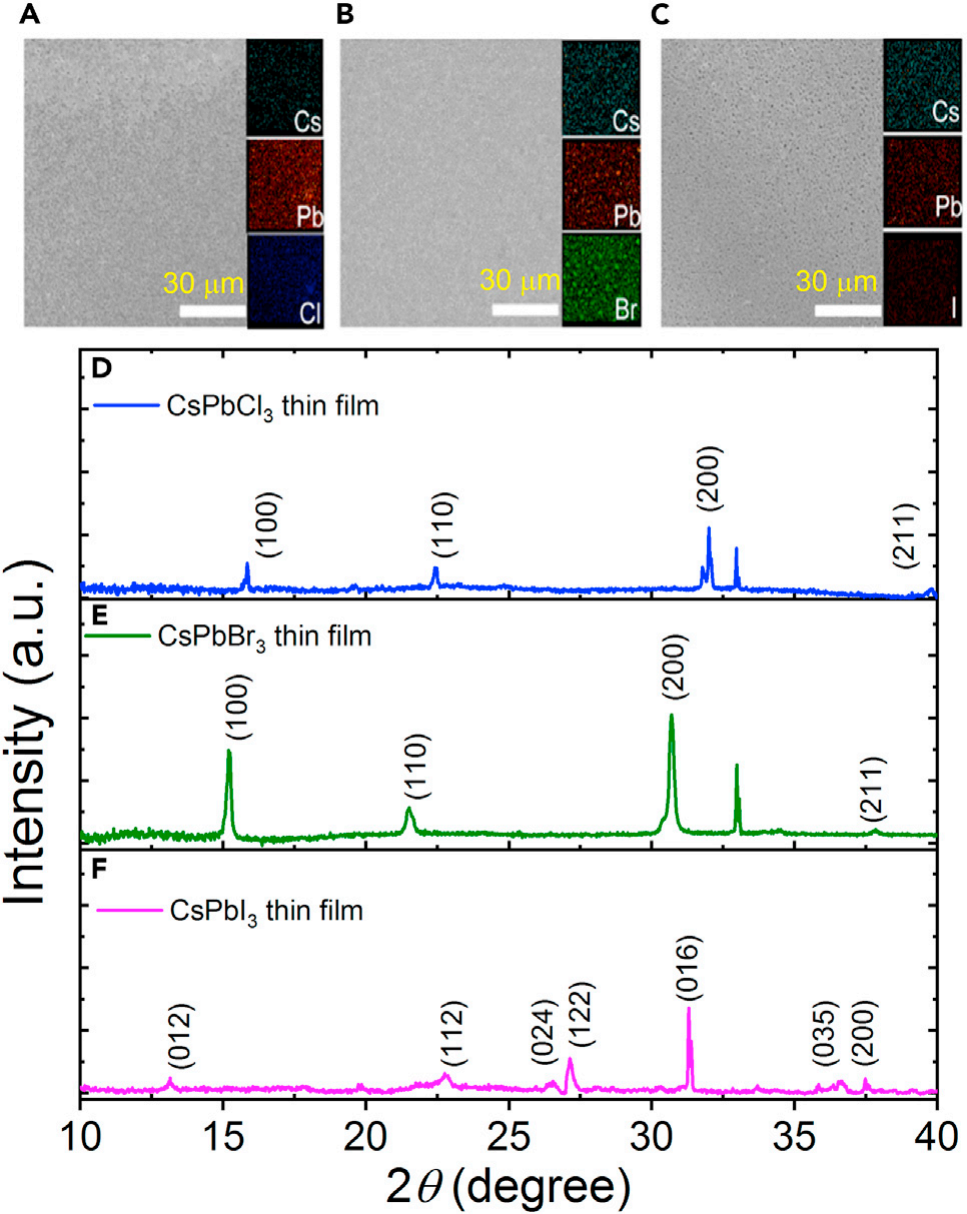
SEM images, EDS and XRD patterns of the typical ternary CsPbX_3_ films (A–C) SEM images and EDS for (A) X = Cl, (B) X = Br, (C) X = I. (D–F) XRD patterns of the CsPbX_3_ (X = Cl, Br and I) thin films.

The all-inorganic perovskite CsPbX_3_ thin films were synthesized and characterized morphologically and optically. The morphology and composition of films are characterized by scanning electron microscopy (SEM) and energy dispersive spectroscopy (EDS). SEM images of the typical ternary CsPbX_3_ films illustrate the thin films with no noticeable variation and low grain boundaries (Figures 2A–2C). The corresponding EDSs are shown at the right sides of Figures 2A–2C. The thickness of all samples was about 200 nm.

The crystal structures of CsPbX_3_ (X = Cl, Br, and I) thin films are characterized by X-ray diffraction (XRD) (Figures 2D–2F). The characteristic XRD peaks (100), (110), (200), and (211) can prove the crystal phase of the CsPbX_3_, which demonstrates that CsPbX_3_ thin films are successfully synthesized. The sharp diffraction peaks indicate that it exhibits good crystallinity.

**Figure 3:**
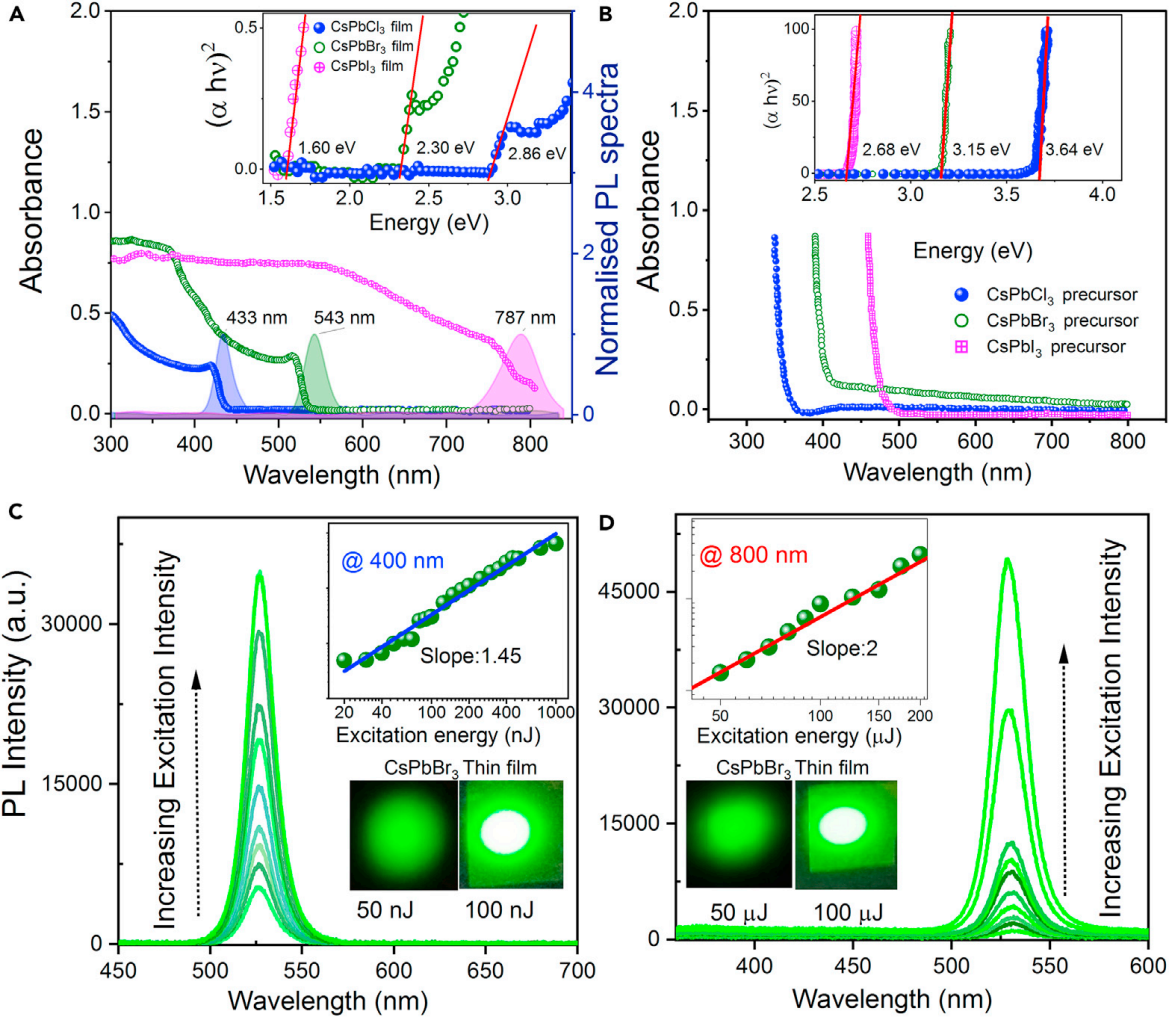
UV-visible and PL spectra of thin films and precursors (A) UV-vis absorption spectra and PL spectra of the CsPbX_3_ (X = Cl, Br, and I) thin films. Inset shows the band gaps of thin films. (B–D) (B) UV-vis absorption spectra of CsPbX_3_ precursors. Inset shows the band gaps of precursors. The measured PL spectra for CsPbBr_3_ at pumping wavelength (C) 400 nm and (D) 800 nm wavelengths for incident laser energies. Top inset shows the tendency of increment in the emitted PL counts, and the Bottom inset shows direct photographs of PL emission from thin film under pulse energy 50 nJ(μJ) and 100 nJ(μJ) in case of 400 nm (800 nm) pumping wavelength.

The ultraviolet-visible (UV-vis) absorption and photoluminescence (PL) spectra of CsPbX_3_ polycrystalline films are shown in Figure 3A, which are the average spectra of 10 measurements. CsPbCl_3_ and CsPbBr_3_ polycrystalline films possess sharp absorption peaks at 418 nm and 516 nm, whereas CsPbI_3_ film demonstrates a broad absorption band from 300 to 800 nm, having a small peak at 752 nm. The emitted PL spectra (excited by 375 nm wavelength) from these thin films confirm the absorption bands. In the case of CsPbCl_3_, CsPbBr_3_, and CsPbI_3_ thin films, the PL spectra peak positions are achieved around 433, 542, and 787 nm, respectively. However, The PL spectra redshifted compared to the absorption peaks.^36^

By considering the absorption spectra (absorption bands), we employed Tauc plot to determine the band gap of the materials. Earlier, several research groups rigorously used the Tauc plots from UV-vis absorption spectra to assess the direct and indirect band gaps of the materials.^23^^,^^37^^,^^38^^,^^39^ The absorption coefficients (α)and the corresponding band gap energies (*E*_g_) of the samples are connected by ${\left(\alpha h\nu \right)}^{n}=A\left(h\nu -E_{g}\right)$ where *h* signifies Planck’s constant, ν denotes photon frequency, and A is a constant. The value of exponent *n* denotes the type of electronic transition in the material. The value n = 2 is for direct authorized transitions, n = 2/3 is for direct forbidden transitions, n = 1/2 is for indirectly allowed transitions, and n = 1/3 is for indirect forbidden transitions.^40^ Nanocrystals, quantum dots, and thin films of CsPbX_3_ perovskites possess direct band gap.^41^^,^^42^^,^^43^^,^^44^^,^^45^^,^^46^ The band gap values are calculated using Tauc plot (*hν verses (hνα)*^*2*^), and the corresponding values for CsPbCl_3_, CsPbBr_3_, and CsPbI_3_ were determined to be 2.85, 2.30, and 1.6, eV, respectively. The band gap of CsPbX_3_ thin films depends on the halide composition and ranges from 1.6 eV to 3.2 eV.^41^^,^^42^^,^^43^^,^^44^ In the present case, the band gap values of thin films are almost similar to those of the reported nanocrystals and quantum dots of the same perovskite materials.^33^^,^^43^^,^^45^^,^^46^

Figure 3B shows the UV-vis absorption spectra for precursors. In the case of precursors, no specific absorption peaks were observed. The absorption edges of precursors are closer to the UV region than those of thin films (Figure 3A). In this case, we also employed the Tauc plot (see inset of Figure 3B) to determine band gaps by considering that the precursors possess direct band gaps (n = 2). The measured band gaps of the precursors (corresponding elements exist in precursors) were 2.68 (CsPbI_3_), 3.15 (CsPbBr_3_), and 3.64 (CsPbCl_3_) eV, respectively.

The differences in the absorption spectra of the films and solutions mainly arise due to the long tail originating from the energy levels of localized defects. As the polarity of the solvent increases, the absorption peak is generated by the *v*-*π*^∗^ transition because of the formation of an H bond between the ground-state *v* electron of the solute molecule and the polar solution. Due to the high polar solution of DMSO, the energy of the ground state is decreased and the ability to form an H bond is increased, causing the blue shift compared to the films. The polarity of solution rises, the absorption spectrum becomes smooth, and the fine structure disappears.

Furthermore, we have studied PL emission properties of CsPbBr_3_ thin film under pumping of femtosecond laser pulses of 400 nm and 800 nm, respectively. The PL emissions with respect to different laser pulse energies are shown in Figures 3C and 3D. The PL peak positions were observed at 526.5 nm and 532 nm, respectively. Since one individual photon energy is lower than the material’s band gap, but the initial excitation energy is higher than the band edge, high nonlinearity will come. The TPA process shows a higher spatial confinement to the focal point of the excitation beam compared to one-photon absorption. It is direct evidence that, under the illumination of 800 nm, the PL emission contributed to TPA for the CsPbBr_3_ thin film. The ratio of the band gap to the excitation photon energy (*E*_g_/*hν*) is equal to 1.48, which lies between 1 and 2. Therefore, the CsPbBr_3_ thin film absorbed two photons and emitted the PL at peak position of 532 nm. Inset of Figures 3C and 3D (top-right/left) shows the tendency of emitted PL peak position counts with respect to laser energies. The linear fits of data points are 1.45 and 2, in the case of 400 nm and 800 nm, respectively. The results indicated that normal luminescence intensity is linearly dependent on excitation power, whereas, under nonlinear conditions, the intensity varies quadratically with pump energy. It is therefore concluded that the luminescence is attributed to TPA under 800 nm.

### NLO response of CsPbX_3_ thin films

The Z-scan data were obtained during the movement of the sample from negative to positive positions regarding the focal plane. We ensured that the nonlinear absorption contributions from the uncoated quartz plate and the empty quartz cell were negligible. The nonlinear absorption could be attributed to various mechanisms, including ground-state bleaching, excited-state absorption, and two- and multi-photon absorption.

Initially, we tried determining the third-order NLO properties for the ∼200 nm thick films at 400 nm and 800 nm excitation wavelengths of 35 fs laser pulses. At these pumping wavelengths, we absorbed the switching of the nonlinear absorption process between reverse saturable absorption (RSA) + saturable absorption (SA) (SA + RSA) for these thin films at different excitation pump intensities.

**Figure 4:**
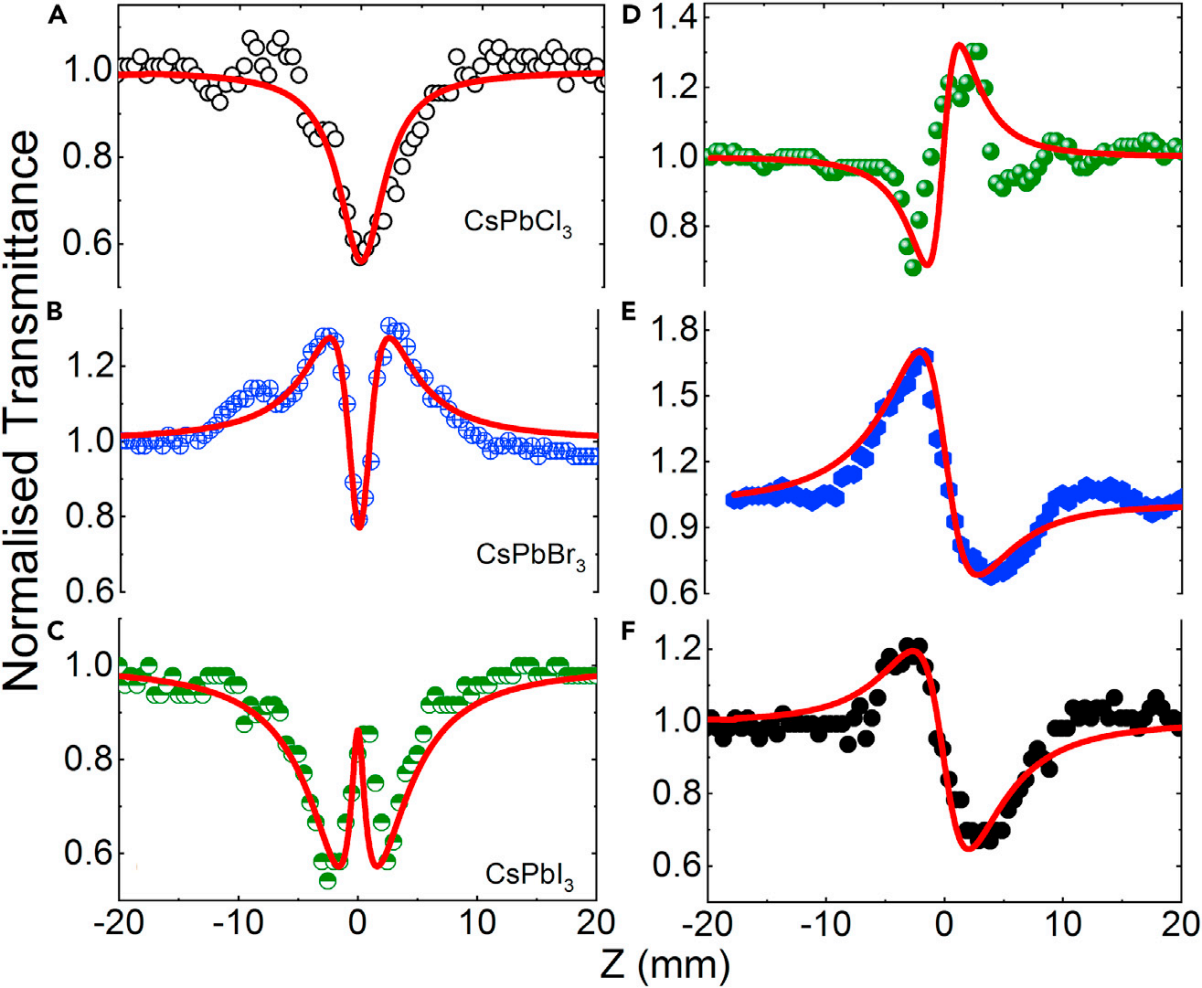
OA and CA Z-scans of CsPbX_3_ films using the 400 nm, 35 fs probe pulses (A–C) OA and (D–F) CA Z-scans of CsPbX_3_ (X = Cl, Br, and I) films. Symbols are the experimental data. The solid lines represent the theoretical fits.

**Figure 5:**
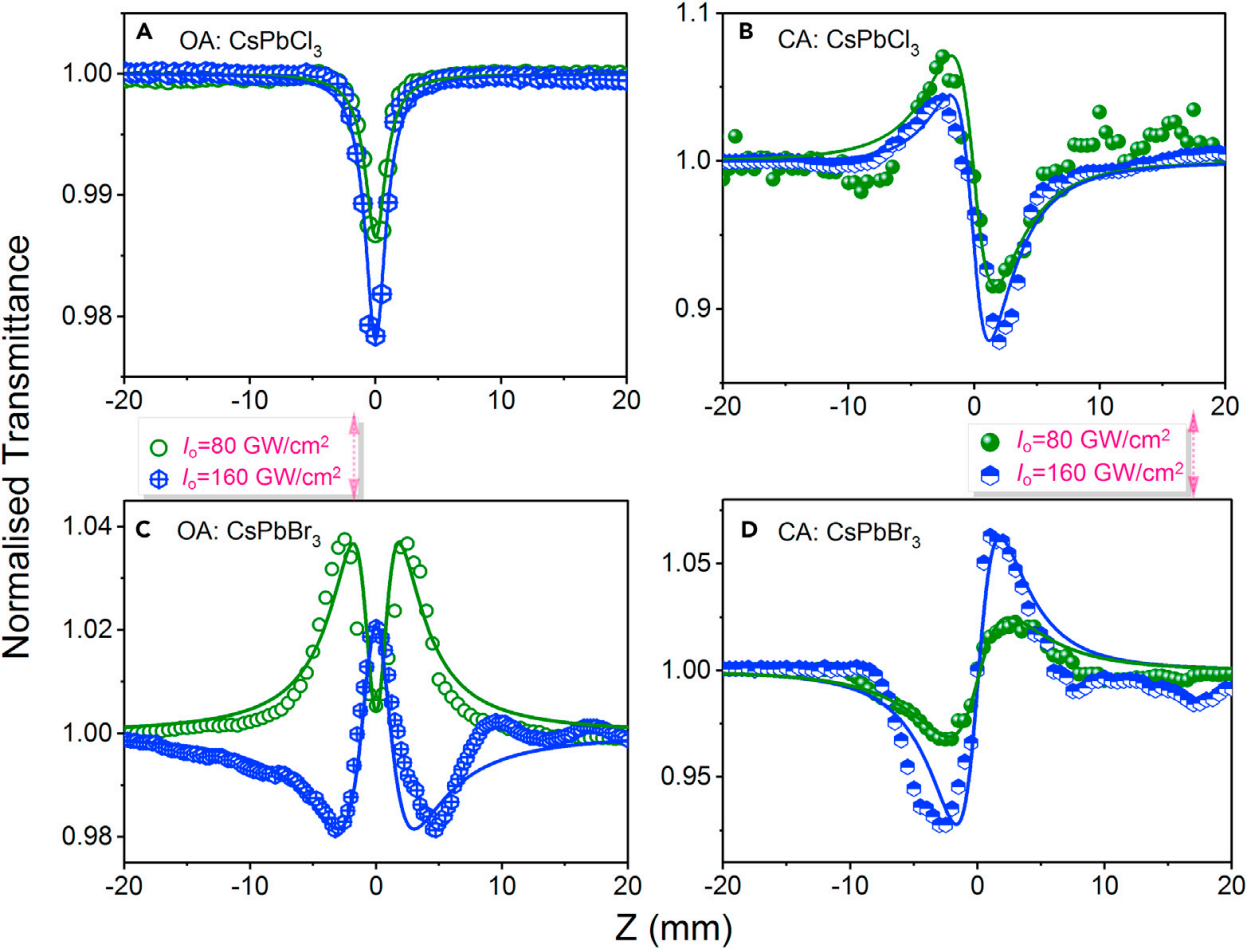
OA and CA Z-scans of CsPbX_3_ films using the 800 nm, 35 fs probe pulses (A and C) OA and (B and D) CA Z-scans of CsPbX_3_ (X = Cl, Br) films using the 800 nm, 35 fs probe pulses. Symbols are the experimental data. The solid lines represent the theoretical fits.

Figures 4A–4F show the opened aperture (OA) and closed aperture (CA) Z-scan data for the CsPbX_3_ thin films using pump wavelength of 400 nm (35 fs pulses) at the peak intensity of 29 GW/cm^2^, whereas, the OA and CA Z-scan data for the CsPbCl_3_ and CsPbBr_3_ thin films of 800 nm (35 fs pulses) at the peak intensity of 80 and 160 GW/cm^2^ are shown in Figures 5A–5D. In both Figures 4 and 5, the solid curves represent the theoretical fits, and the calculated values of *β*, *γ*, and *I*_sat_ are summarized in the Table 1.

**Table 1:** Summary of NLO parameters for perovskite thin films

Thin film	*λ* (nm)	*I*_*0*_*GW/cm*^*2*^	*β* (cm/W)	*γ* (cm^2^/W)	Im*χ*^*3*^ (esu)	Re*χ*^*3*^ (esu)	*|χ*^*3*^| (esu)	*I*_sat_ (W/cm^2^)
CsPbCl_3_	400	29	−1.9 × 10^−6^	7.4 × 10^−10^	−3.4 × 10^−6^	4.2 × 10^−8^	3.4 × 10^−6^	*-*
CsPbBr_3_	400	29	3.9 × 10^−6^	−1.4 × 10^−11^	6.9 × 10^−6^	−8 × 10^−10^	6.9 × 10^−6^	2.8×10^10^
CsPbI_3_	400	29	3.6 × 10^−5^	−6.4 × 10^−10^	6.4 × 10^−5^	−3.7 × 10^−8^	6.4 × 10^−5^	3.4×10^10^
CsPbCl_3_	800	80	2.4 × 10^−8^	−3.1 × 10^−12^	9.1 × 10^−10^	−1.8 × 10^−6^	1.8 × 10^−6^	–
	800	160	1.9 × 10^−8^	1.6 × 10^−12^	7.3 × 10^−10^	−9.3 × 10^−8^	9.3 × 10^−8^	–
CsPbBr_3_	800	80	2.7 × 10^−7^	1.1 × 10^−12^	1.0 × 10^−8^	6.5 × 10^−7^	6.5 × 10^−7^	8.0×10^10^
	800	160	1.4 × 10^−8^	1.3 × 10^−12^	5.2 × 10^−9^	7.7 × 10^−7^	7.7 × 10^−7^	1.59×10^11^

The OA Z-scans shown in Figures 4A–4C were obtained using the same intensity at the focus. The pump photon energy was 3.1 eV. It was measured that these films show the band gaps 1.6, 2.3, and 2.86 eV for the CsPbI_3_, CsPbBr_3_, and CsPbCl_3_, respectively. The CsPbI_3_ film may possess RSA+SA or TPA+SA. The pump photon has larger energy than the band gap of thin films. Therefore, the SA occurred in these thin films as expected. However, the CsPbCl_3_ thin film shows RSA due to the higher band gap than the other two. The role of RSA+SA or TPA+SA in the CsPbBr_3_ and CsPbI_3_ thin film is involved in the complexity of higher energy states as we distinguished. It is understood that initially the film absorbs two photons due to TPA, and the electrons jump to deeper conduction bands. Then, once the peak intensity of the pump laser pulse reaches to the maximum, SA starts playing an important role alongside the strong positive nonlinear absorption.

Meanwhile, the CsPbBr_3_ film demonstrated SA + RSA at 400 nm pumping (29 GW/cm^2^) (see Figure 4B). The same combination of NLO absorption processes was reported in the case of CsPbBr_3_ nanocrystals with an average size of ∼20 nm in the case of excitation using similar photons as in our case (3.1 eV, 400 nm).^47^ In the case of the weak intensity of pump pulses, SA first appears (due to the ground-state bleaching of sp^2^ domains and the depletion of the valence band). Then, at higher intensities, the switch from SA to RSA occurs due to the involvement of higher energy levels of the studied sample. However, in the case of 800 nm pulses, CsPbBr_3_ thin film possess SA + RSA at 80 GW/cm^2^, and the absorption phenomena switched to RSA(TPA)+SA at a higher intensity of 160 GW/cm^2^ as shown in Figure 5C.

**Figure 6:**
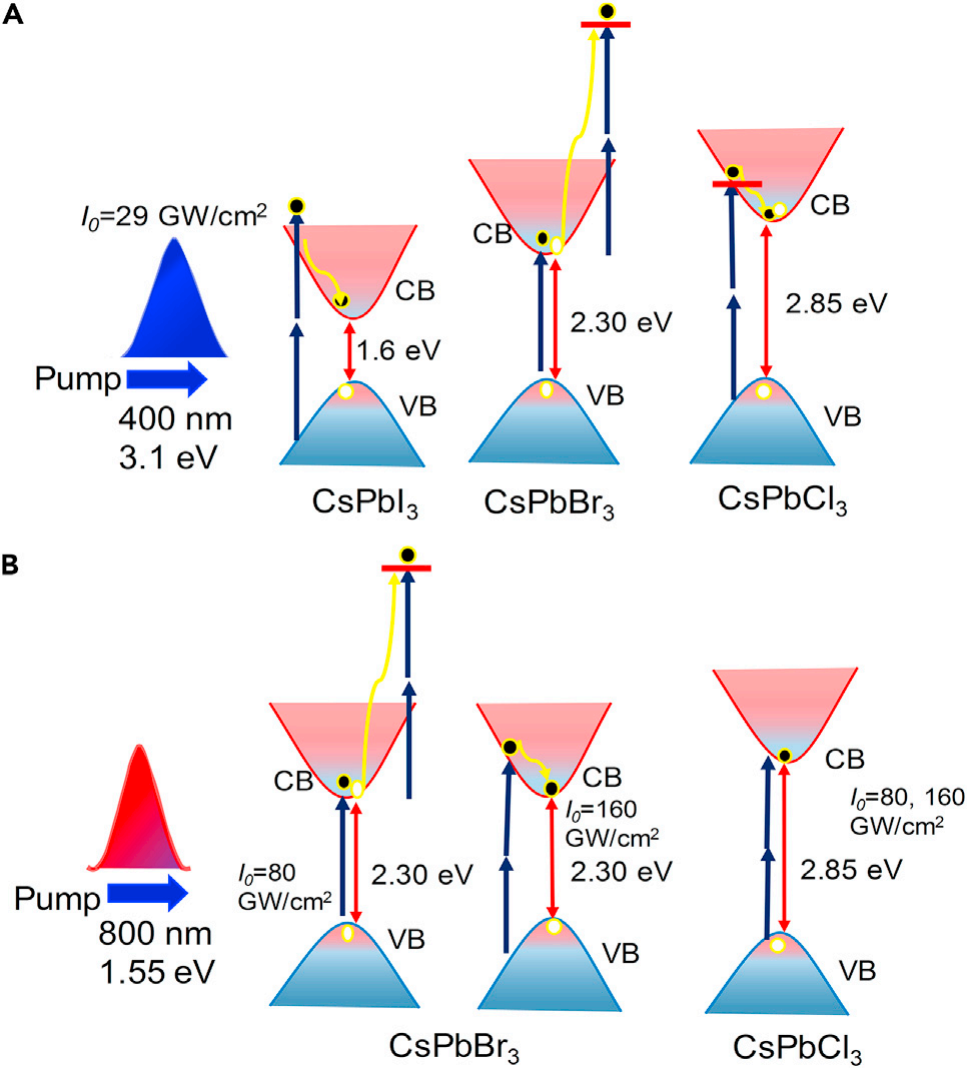
Simplified energy level diagram Indicating the states and transitions involved in saturable absorption and two-photon absorption between valence band (VB) and conducting band (CB) at pump wavelength (A) 400 nm and (B) 800 nm, respectively.

The CsPbCl_3_ films demonstrate either RSA or TPA at both pumping wavelengths (400 nm and 800 nm) as shown in Figures 4A and 5A, due to the band gap of CsPbCl_3_. As shown in Figure 5A, the absorption dip is increased at 160 GW/cm^2^ compared to 80 GW/cm^2^; however, the nonlinear absorption coefficient is decreased in the former case due to the higher intensity of pumping wavelength. In this case, one can expect that the pump photon energy contributes to absorption of two photons to allow the valence-band electrons to jump to the conduction band. The schematic of the nonlinear absorption of thin films at pump energy 3.1 eV and 1.5 is shown in Figure 6. It is observed that SA plays a dominant role in the case of the CsPbBr_3_ thin film at both 400 nm and 800 nm wavelengths and CsPbI_3_ thin films at 400 nm pulses.

The upper valence band and the lower conduction band of the perovskites are formed predominantly by the halide *p*-orbitals and the Pb *p*-orbitals, respectively. For the semiconducting resonant band, *I*_*sat*_ is lower for the nearer resonant peak.^48^ Notice that the morphology of perovskite and the decrease of interstitial Pb atoms can also affect the NLO properties of these films. The morphology control provides optimal coverage of thin films and the formation of the smaller perovskite crystals. Therefore, decreasing the distance between grains in polycrystalline films increases the multiple scattering. Correspondingly, it increases the effective interaction length and, consequently, the nonlinear absorption.

CA Z-scan measurements at the wavelength of 400 nm revealed a self-focusing of CsPbCl_3_ film and self-defocusing of CsPbBr_3_ and CsPbI_3_ films (see Figures 4D–4F), whereas, at pumping wavelength 800 nm CsPbCl_3_ film possess self-defocusing (Figure 5B) and CsPbBr_3_ film shows self-focusing (see Figure 5D). If we could compare the CsPbCl_3_ and CsPbBr_3_ thin films at each pump wavelength, they possess opposite focusing effects. The nonlinear Kramers-Kronig relations predict the self-defocusing in semiconductors for which the relation *ħω*/*E*_g_ > 0.69 takes place.^49^ Here *ħ* is the Planck’s constant, *ω* is the frequency of laser radiation, and *E*_g_ is the band gap energy of the semiconductor (2.3 and 2.86 eV in the case of CsPbBr_3_ and CsPbI_3_ films, respectively). The corresponding *ħω*/*E*_g_ values for these films at *λ* = 400 nm (*ħω* = 3.1 eV) were calculated to be 1.35 and 1.08, respectively. Thus, one can expect the negative sign of *γ* in these two films that was confirmed in our experiments. As for the CsPbCl_3_ films, the value of *ħω*/*E*_g_ at *λ* = 400 nm is equal to 1.86, which means that this film should also demonstrate the self-defocusing properties, contrary to our observations of the positive sign of *γ* in this thin film. One of the assumptions here is the involvement of the higher-order positive NLO refraction. This assumption is based on the smaller distance between peak and valley of CA curve along the z axis in the case of CsPbCl_3_ film (∼3 mm, Figure 4D) compared with other films (∼5 mm, Figures 4E and 4F), which is a sign of involvement of the higher-order NLO process. However, the self-defocusing effect was observed in the case of 800 nm (*ħω* = 1.55 eV) pumping for CsPbCl_3_ thin film. The corresponding *ħω*/*E*_g_ value is 0.96. Thus, the observed self-focusing/self-defocusing in the CsPbCl_3_ perovskite films at 400nm/800 nm requires additional analysis.

The difference in the nonlinear absorption properties of polycrystalline films may result from the additional synergistic effect that comes from the interaction between the halogen atom and metal atom. With the input power of 60 μW, a transition from the transmission peak to the transmission valley can be observed for the CsPbBr_3_ film.

From the aforementioned results, we can see two types of nonlinear absorption with opposite signs in perovskite films in the case of the 400 nm, 35 fs probe pulses. The intensity-dependent linear absorption coefficient is presented by the following equation:

(Equation 1) $$\frac{dI}{dz}={-\alpha }_{0}I-\beta \left(I\right)I^{2}$$

where $\alpha_{0}$ is the linear absorption coefficient of the medium and β(I) is the nonlinear absorption coefficient, which can depend on the intensity of the laser pulses. The nonlinear absorption coefficient is negative in the case of SA and positive in the case of TPA. The SA can occur in semiconductors where the excitation of electrons from the valence band into the conduction band reduces the absorption for photon energies just above the band gap energy. The SA effect can be described using different models.

We analyzed the non-stationary model for explanation of TPA and SA in CsPbI_3_. A simple hyperbolic approximation was used to model the intensity variation. To interpret the flip toward SA in the vicinity of the focal plane (Figure 4C), we phenomenologically combined an SA coefficient and a TPA coefficient, yielding the total absorption coefficient as^50^

(Equation 2) β(I)=β01+IIsat

where *β*_0_ is the TPA-induced low intensity response of the material and *I*_sat_ is the saturation intensity at which *β*_0_ is divided by 2. If the band gap of material is equal to the energy of photon of the probing pulses, then the nonlinear process can be described as the following equation.

(Equation 3) β(I)=α0+β01+(IIsat)2

The hyperbolic model corresponds to the single-photon SA in CsPbBr_3_, where the value of band gap energy is close to the energy of the photon energy of probing pulses. In the case of CsPbBr_3_, we observed a single-photon SA at the low intensity of input pump (out of focal plane, Figure 4B). At peak intensity (near to focal area) the TPA (or most probably RSA) dominates due to excited states free carrier absorption.^23^ The electrons excited via the interband transitions of CsPbCl_3_ and CsPbI_3_ and referred to as free carriers have a whole spectrum of energies, including kinetic and potential energies. The potential energies arise from the formerly unoccupied and occupied states within the conduction band.

### NLO response of CsPbX_3_ precursors

**Figure 7:**
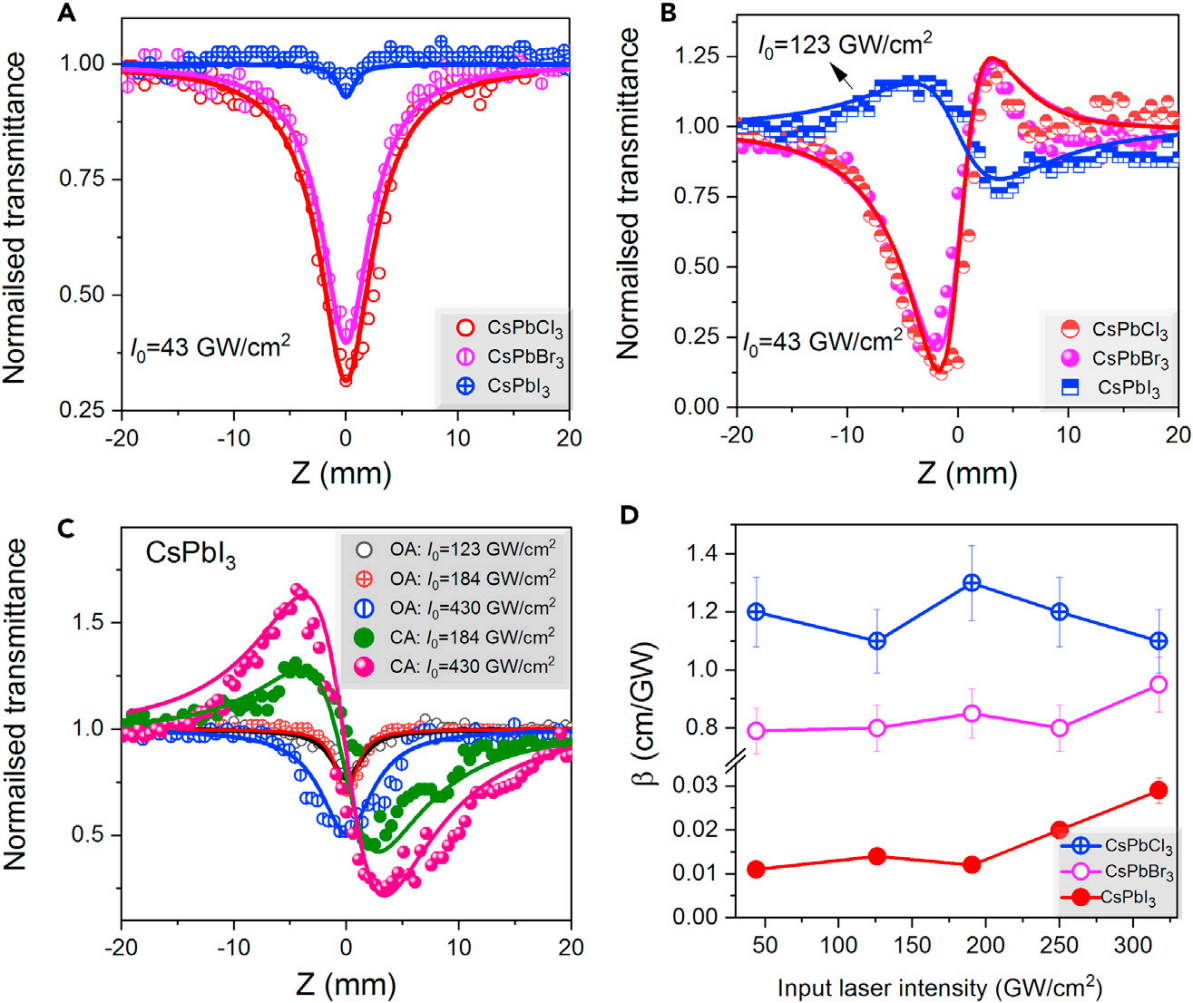
Z-scans using 800 nm, 35 fs pulses for CsPbX_3_ precursors (A) OA data, at 43 GW/cm^2^. (B) CA data. (C) OA and CA data for CsPbI_3_. The solid curves in (A–C) correspond to the theoretical fits. (D) The variations of *β* of three precursors at different intensities of probe pulses.

Figure 7A shows the OA Z-scans of CsPbCl_3_, CsPbBr_3_, and CsPbI_3_ precursors using the 800 nm, 35 fs pulses at *I*_0_ = 43 GW/cm^2^. These curves show the minimum transmittance attributed to the TPA in these three perovskite precursors. CsPbCl_3_ and CsPbBr_3_ showed strong TPA, while CsPbI_3_ demonstrated notably smaller nonlinear absorption, which is due to its lower band gap (2.68 eV, *E*_800 nm_ = 1.55 eV) compared to the CsPbCl_3_ (3.64 eV) and CsPbBr_3_ (3.15 eV) precursors.

Figure 7B depicts the CA Z-scan curves showing that CsPbCl_3_ and CsPbBr_3_ precursors exhibit positive nonlinear refraction at 43 GW/cm^2^, whereas, in the case of CsPbI_3_ precursor, the almost three times higher intensity (i.e., 123 GW/cm^2^) required for observing the NLO effect shows that this suspension possesses the self-defocusing effect. Consequently, for this precursor, the increase of intensity of laser pulses (i.e., 183 and 430 GW/cm^2^) showed the growth of self-defocusing during CA measurements (see Figure 7C). The magnitude of *γ* of precursors at *λ* = 800 nm was calculated by fitting the CA data using Equation 7, and the obtained values were in the range of 10^−15^ to 10^−16^ cm^2^ W^−1^ (see Table 2, where all NLO parameters of precursors are collected).

**Table 2:** Summary of NLO measurements of the CsPbX_3_ precursors

Precursor	NLO parameter	Pump wavelength (nm)			
		355	400	800	1064
CsPbCl_3_ CsPbBr_3_ CsPbI_3_	*β* (cm/W)	6.1 × 10^−8^ 5.3 × 10^−7^ ^-^	6.2 × 10^−11^ 8 × 10^−10^ 8.6 × 10^−10^	3.0 × 10^−11^ 2.7 × 10^−11^ 2.5 × 10^−11^	2.5 × 10^−9^ 5 × 10^−10^ 6.9 × 10^−11^
CsPbCl_3_ CsPbBr_3_ CsPbI_3_	*γ*(cm^2^/W)	7.9 × 10^−14^ −2.3 × 10^−12^ –	6 × 10^−16^ 3 × 10^−15^ 8.8 × 10^−16^	4.7 × 10^−16^ 3.2 × 10^−16^ −6.5 × 10^−16^	5.1 × 10^−14^ −3.6 × 10^−14^ −4.7 × 10^−14^
CsPbCl_3_ CsPbBr_3_ CsPbI_3_	Im*χ*^*3*^(esu)	9.6 × 10^−8^ 8.3 × 10^−7^ –	1.1 × 10^−10^ 1.4 × 10^−9^ 1.5 × 10^−9^	1.1 × 10^−10^ 9.5 × 10^−11^ 8.8 × 10^−11^	1.2 × 10^−8^ 2.3 × 10^−9^ 3.2 × 10^−9^
CsPbCl_3_ CsPbBr_3_ CsPbI_3_	Re*χ*^*3*^(esu)	4.5 × 10^−12^ −1.3 × 10^−10^ –	3.4 × 10^−14^ 1.7 × 10^−13^ 5 × 10^−14^	2.7 × 10^−14^ 1.8 × 10^−11^ 3.7 × 10^−14^	2.9 × 10^−12^ −2.1 × 10^−12^ −4.7 × 10^−12^
CsPbCl_3_ CsPbBr_3_ CsPbI_3_	*|χ*^*3*^|(esu)	9.6 × 10^−8^ 8.3 × 10^−7^ –	1.5 × 10^−9^ 1.4 × 10^−9^ 1.1 × 10^−10^	1.1 × 10^−10^ 9.5 × 10^−11^ 8.8 × 10^−11^	1.1 × 10^−10^ 2.3 × 10^−9^ 3.2 × 10^−9^

To illustrate the mechanism of nonlinear absorption of studied precursors, the obtained *β* values as the functions of input intensities are shown in Figure 7D. With the increase of laser intensity, the *β* of three precursors remains approximately same, thus indicating that TPA dominates among other nonlinear absorption processes in these precursors at the wavelength of 800 nm and 35 fs pulse duration.

**Figure 8:**
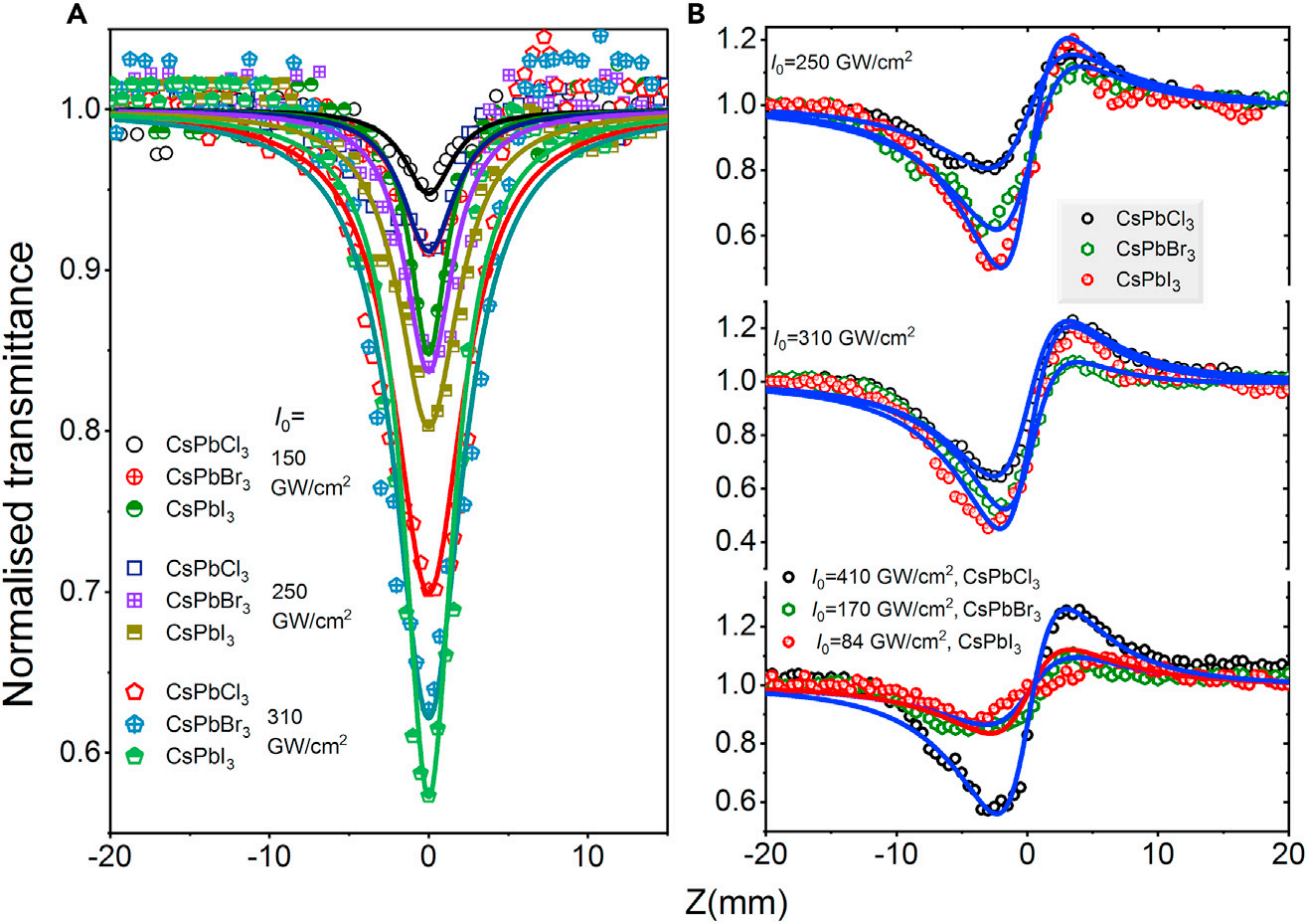
Z-scans of CsPbX_3_ precursors using 400 nm, 35 fs pulses (A) Open aperture Z-scan data at 150, 250, and 310 GW/cm^2^, (B) Closed aperture Z-scan data (upper panel: 250 GW/cm^2^, middle panel: 350 GW/cm^2^, bottom panel: 410 GW/cm^2^ for CsPbCl_3_, 170 GW/cm^2^ for CsPbBr_3_ and 84 GW/cm^2^ for CsPbI_3_).

The modulation depth of Z-scan curves in the case of the resonant wavelength (400 nm) should be enhanced compared with the 800 nm non-resonant wavelength, as expected from the previous studies.^51^^,^^52^ Figure 6 shows the OA and CA Z-scan data for CsPbX_3_ precursors at 400 nm (*E*_400 nm_ = 3.1 eV) at different input intensities of laser pulses. In this case, the RSA was observed for three precursors. Figure 8A depicts three OA curves; among them the RSA is stronger for CsPbI_3_ precursor and followed by RSA _CsPbBr3_ > RSA _CsPbI3_. Figure 6B (upper panel and middle panel) shows the CA curves measured at 250 and 310 GW/cm^2^ intensities of 400 nm pulses, respectively. In the case of CA data, the values of dip are higher than in the case of other two precursors due to strong absorption of CsPbI_3_. At 400 nm excitation, all precursors possess self-focusing effect, unlike the 800 nm case.

Figure 8B (bottom panel) presents the CA data at lower intensity (84 GW/cm^2^) for CsPbI_3_, moderate intensity (170 GW/cm^2^) for CsPbBr_3_, and high intensity (410 GW/cm^2^) for CsPbI_3_. Notice that in the case of thin films excited by 400 nm pulses the nonlinear absorption and refraction vary for three samples, while in the case of precursors we did not observe the variations of these processes.

**Figure 9:**
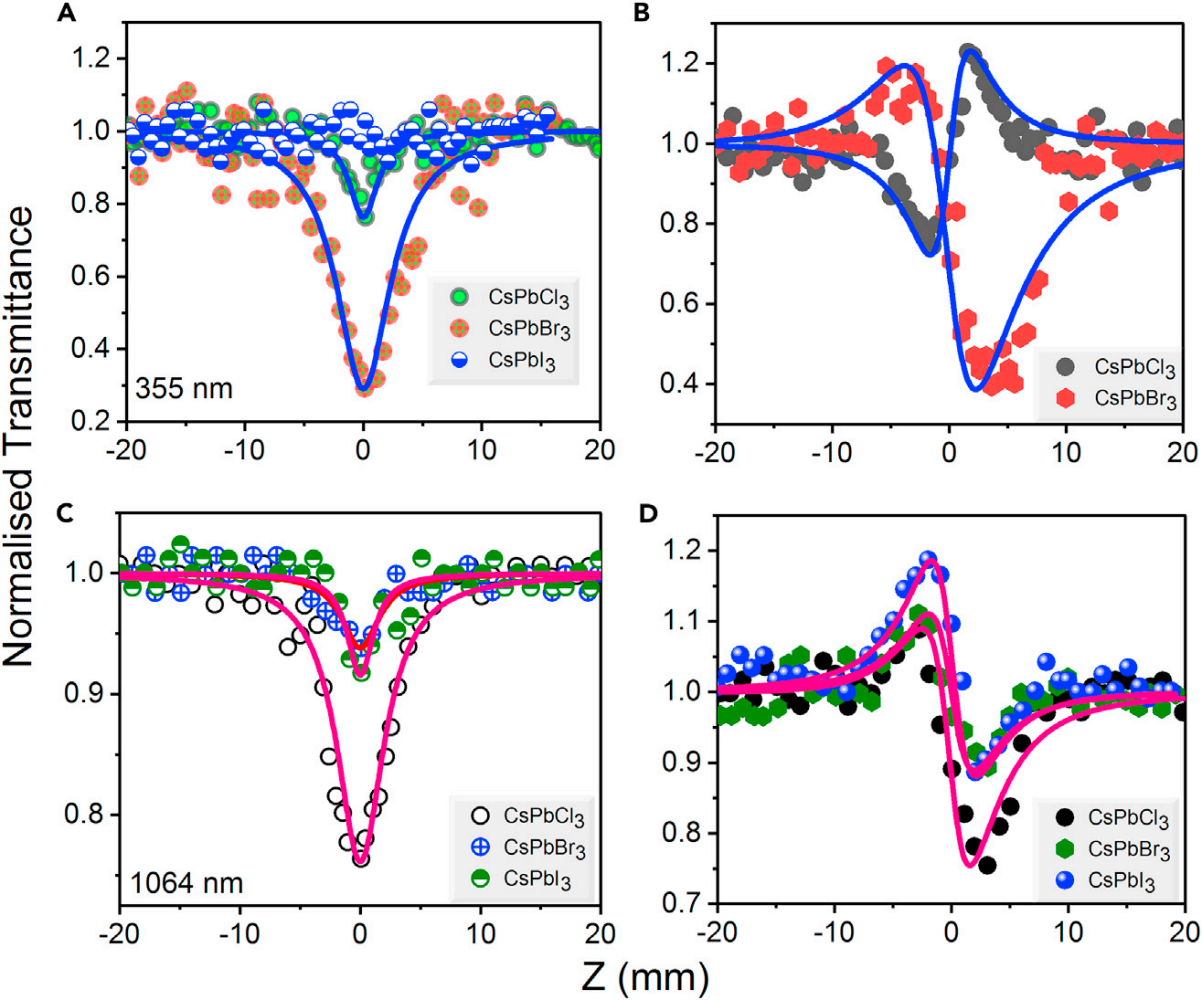
OA and CA Z-scans of CsPbX_3_ (X = Cl, Br, I) precursors In the case of using (A and B) 355 nm (*I*_0_ = 4.7 GW/cm^2^) and (C and D) 1,064 nm (*I*_0_ = 8.1 GW/cm^2^), 6 ns pulses.

Symmetric transmission curves shown in Figure 9 are Z-scans measured at 6 ns pulses, wavelengths of 355 nm and 1,064 nm and laser energy of 70 μJ and 120 μJ, respectively. The corresponding peak intensities are 4.7 GW/cm^2^ and 8.1 GW/cm^2^, respectively. The CsPbI_3_ precursor does not possess any nonlinear absorption at 355 nm until the sparkling occurs (Figure 9A) at *I*_0_ = 4.7 GW/cm^2^. It is expecting that the CsPbI_3_ solution demonstrates the linear transmittance during propagation of 355 nm pulses through the focal region. However, we tried to increase the laser energy to test the CsPbI_3_ solution. The CsPbI_3_ solution shows the glint when we increase the laser energy due to high photon energies of 355 nm. Therefore, we optimized the same laser energy for 355 nm pumping at 70 μJ to compare three solutions. Among CsPbBr_3_ and CsPbCl_3_, the first precursor shows strong RSA at this pump wavelength. Meanwhile, the nonlinear refraction also absents in case of CsPbI_3_, whereas CsPbBr_3_ possess self-defocusing and CsPbCl_3_ shows self-focusing effects at 355 nm (see Figure 9B). Figures 9C and 9D shows the OA and CA Z-scans for excitation wavelength 1,064 nm. In that case, CsPbCl_3_ precursor demonstrates stronger nonlinear absorption and refraction than the other two precursors due to the lower band gap of CsPbCl_3_ (2.68 eV) and lower photon energy (1.16 eV).

**Figure 10:**
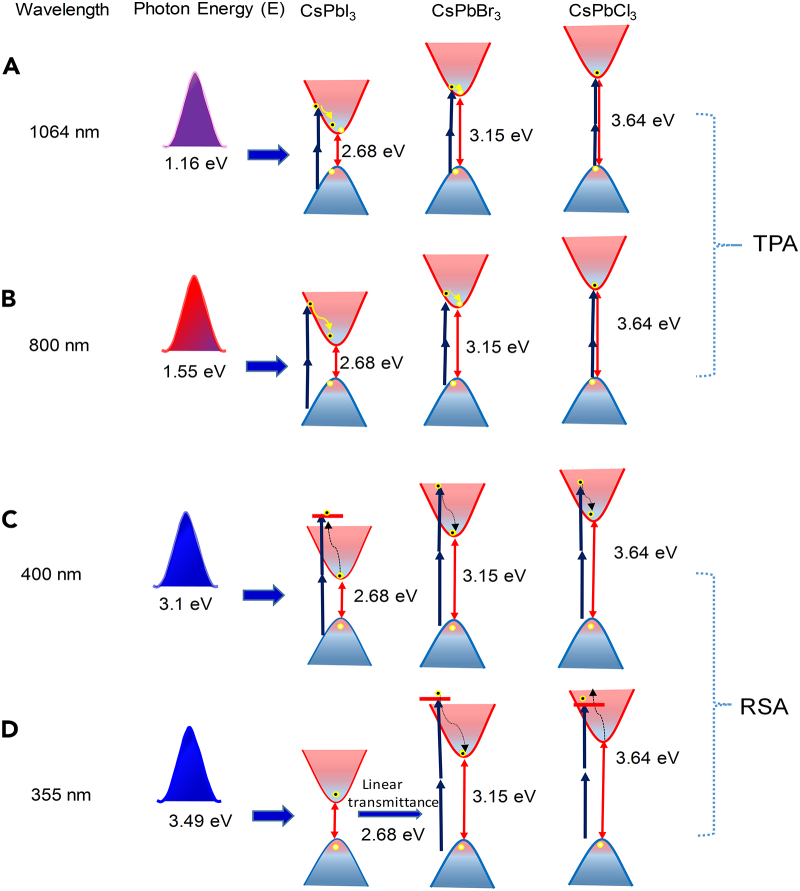
Schematic representation of energy levels for TPA and RSA processes in CsPbX_3_ precursors At (A) 1,064 nm, (B) 800 nm, (C) 400 nm, and (D) 355 nm, wavelengths of pump radiation.

Overall, in the case of three precursors either TPA or RSA plays a pivotal role depending on the pumping photon energy (1.16 or 3.49 eV). Among the three precursors, the level of nonlinear absorption processes at different pumping wavelengths is summarized in Table 3. The typical energy level diagrams for these precursors to represent the TPA at 1,064 nm and 800 nm and RSA at 400 nm and 355 nm are shown in Figures 10A–10D, respectively.

**Table 3:** The relation between the nonlinear absorption responses of precursors at different wavelengths

Wavelength (nm)	Photon energy (eV)	Nonlinear absorption order
1064	1.165	CsPbCl_3_> CsPbBr_3_> CsPbI_3_
800	1.55	CsPbCl_3_> CsPbBr_3_> CsPbI_3_
400	3.10	CsPbI_3_> CsPbBr_3_> CsPbCl_3_
355	3.49	CsPbBr_3_> CsPbCl_3_ No absorption in the case of CsPbI_3_

Notice that, in the case of precursors, TPA (or RSA) dominates over other NLO processes at all used wavelengths, while SA plays the dominant role in the case of the CsPbBr_3_ and CsPbI_3_ thin films probed by 400 nm pulses and CsPbBr_3_ thin film probed by 800 nm pulses. Like precursors, CsPbCl_3_ thin films shows TPA or RSA at 800 nm and 400 nm pulses due to its larger band gap (2.85 eV) compared to the other two films. If we consider that the band gaps of all precursors vary from 2.68 to 3.64 eV, the CsPbCl_3_ thin film band gap lies between in this range. Therefore, at pumping wavelength energy between 1.16 eV and 3.49 eV in the case of precursors and at 3.1 eV and 1.55 eV, pump energies for CsPbCl_3_ thin films show the TPA (or RSA). Perovskite precursor solutions are generally composed of stoichiometric mixtures of PbX_2_ and CsX (X = Cl, Br, and I) in solvents, which makes it difficult to know the stoichiometry and structure in the perovskite precursor solution. Moreover, thin film has crystal morphology and perovskite crystal orientation (confirmed by XRD data), which enhances the NLO properties and reversibility of the nonlinear absorption process, either SA + RSA or SA + TPA.

### Conclusion and outlook

In conclusion, we have investigated third-order NLO properties of inorganic perovskite CsPbX_3_ (X = Cl, Br, I) precursors at the wavelengths of 355 nm, 400 nm, 800 nm, and 1,064 nm and pulses of different durations (6 ns and 35 fs) and thin films at the 400 nm and 800 nm, 35 fs pulses using the Z-scan technique. Due to the polycrystalline nature of thin films and having specific direct band gaps, thin films possess nonlinear absorption processes such as TPA, TPA+SA, and SA + RSA. At the same time, it was shown that the RSA and TPA play pivotal roles in the studied precursors. The nonlinear refraction was analyzed in the precursors and films. The obtained NLO parameters for thin films at 400 nm and 800 nm showed higher values than the precursors. The thin films exhibited superior nonlinearities at 400 nm (*β* = 3.6 × 10^−5^ cm W^−1^, *γ* = 7.4 × 10^−10^ cm^2^ W^−1^). However, the nonlinear absorption coefficient and nonlinear refraction index were significantly changed from one to another precursor. The strong NLO parameters in the ultraviolet range make such materials suitable for the imaging and photonic device applications.

### Limitations of the study

The current study focused on retrieving the third-order NLO properties in different incident pump wavelengths, which is an effective means for expanding the application range of the perovskites in nonlinear optics. Despite the superior NLO properties, lead toxicity and low chemical stability of these perovskites remain a concern, which requires exploring new methods to alleviate these issues.

## STAR★Methods

### Key resources table

**Table:** 

REAGENT or RESOURCE	SOURCE	IDENTIFIER
**Chemicals, peptides, and recombinant proteins**		

CsCl	Sigma-Aldrich	CAS#7647-17-8
CsBr	Sigma-Aldrich	CAS#7787-69-1
CsI	Sigma-Aldrich	CAS#7789-17-5
PbCl_2_	Sigma-Aldrich	CAS#7758-95-4
PbBr_2_	Sigma-Aldrich	CAS#10031-22-8
PbI_2_	Sigma-Aldrich	CAS#10101-63-0
Dimethyl sulfoxide (DMSO)	Sigma-Aldrich	CAS#67-68-5

**Others**		

Ti: Sapphire laser, 35 fs, 1 kHz	USA	Spitfire Ace, Spectra-Physics
ND: YAG laser	USA	Q-smart, Coherent.
UV-vis-NIR spectrometer	USA	Varian Cary 5000
scanning electron microscope	Japan	SEM, Hitachi, S-4800
confocal laser Raman spectrometer	Germany	RenishawinVia-Reflex
X-ray diffractometer	Germany	Bruker, D8 focus

**Software and algorithms**		

Inkscape	Open source	https://inkscape.org
Origin Pro	Open source	https://www.originlab.com/

### Resources availability

#### Lead contact

Further information and requests for resources should be directed to and will be fulfilled by lead author, Wei Li (weili1@ciomp.ac.cn (W.L)).

#### Materials availability

This study did not generate new unique reagents. All chemicals were obtained from commercial resources and used as received.

#### Data and code availability

• Data: All data reported in this paper will be shared by the lead contact upon request.
• Code: This study does not generate a new code.
• Additional information: Any additional information required to reanalyze the data reported in this study is available from the lead contact upon request.

### Experimental model and study participant details

#### Characterization of samples

UV-vis absorption spectra were recorded at room temperature using a UV-vis-NIR spectrometer (Varian Cary 5000). The photoluminescence (PL) emission spectra were analyzed by a microscopic confocal laser Raman spectrometer (Renishaw inVia-Reflex). The morphology and elemental distribution of polycrystalline thin films were studied using a scanning electron microscope (SEM, Hitachi, S-4800, Japan). The crystallinity orientation of X-ray diffraction (XRD) of CsPbX_3_ was illustrated by an X-ray diffractometer (Bruker, D8 focus, Germany).

#### Z-scan and PL arrangements

For the NLO measurements, we used 800 nm (Ti: Sapphire laser, 35 fs, 1 kHz, Spitfire Ace, Spectra-Physics), and 400 nm (obtained from fundamental radiation using the frequency doubling in barium borate (BBO) crystal) radiation, as well as 1064 nm and 355 nm, 6 ns pulses delivered from Nd: YAG laser (Q-smart, Coherent, USA). The samples were placed on a translation stage, and their optical transmittance was collected using a photodiode and lock-in amplifier. The experimental schematic of the Z-scan technique is shown in Figure 1. In brief, the laser beam propagated through the sample and then collected by a photodiode behind the opened aperture (OA) or closed aperture (CA). The beam was focused on the sample using a 400 mm focal length convex lens in measurements of thin films at 400 nm and precursors at 1064 nm, 800 nm, 400 nm, and 355 nm, whereas for thin films at 800 nm excitation, we used the 200 mm lens. The beam waist diameters were measured using a sensor and found to be 38 μm and 16 μm, corresponding to 400 mm and 200 mm lens, respectively. The computer-controlled translational stage was used for scanning the samples along the z axis.

For the PL arrangements, the CsPbBr_3_ thin film was kept in the middle of 200 mm focusing lens and focus spot along the laser propagation path. The 800 nm pulses power was controlled by an attenuator and 400 nm pulses were generated by the same BBO crystal used for Z-scan measurements, unconverted 800 nm was blocked by color filter, as shown in the top middle of Figure 1. The laser pulses were illuminated on thin film at desired energies. The emitted PL light was collected by USB spectrometer ranging from 200 nm to 1100 nm.

The beam was focused on the sample using a 400 mm focal length convex lens. The beam waist diameter was measured using a sensor and found to be 38 μm.

#### Basic relations of Z-scans

OA and CA- Z-scans were employed to study the nonlinear absorption (NLA) and nonlinear refraction (NLR) of CsPbX_3_ (X = Cl, Br, I) precursor solutions and films. The normalized transmittances in the case of two kinds of nonlinear absorption (TPA and saturable absorption (SA)) in the case of the OA Z-scan are given by^53^^,^^54^^,^^55^^,^^56^^,^^57^

(Equation 4) $$T\left(z\right)=q^{-1}\times \ln\left(1+q\right)$$

(Equation 5) $$T\left(z\right)=1+\frac{I_{0}}{I_{sat}\left(x^{2}+1\right)}$$

Here *I*_sat_ is a saturation intensity of SA, *x = z/z*_0_ is a relative coordinate, *q* = *βI*_0_*L*_eff_/(1 + *z*^2^/*z*_0_^2^), *z*_0_ *= k(w*_0_*)*^*2*^*/2* is a Rayleigh length, *β* is a nonlinear absorption coefficient, *k = 2π/λ* is a wave number, *w*_0_ is a beam waist radius at the 1/e^2^ level of intensity distribution, *I*_0_ is an intensity in the focal plane, *L*_eff_ *=* [1-exp(-*α*_0_*L*)]/*α*_0_ is an effective length of the medium, *α*_0_ is a linear absorption coefficient, and *L* is a thickness of studied samples.

CA Z-scan allows determining NLA and NLR when they are presented simultaneously. In general case of the joint contribution of both those processes, the normalized transmittance of samples along z axis, *T*(z), can be presented as follows^53^^,^^55^

(Equation 6) $$T\left(z\right)=1+\frac{4x}{\left(x^{2}+9\right)\left(x^{2}+1\right)}\Delta {Ф}_{0}-\frac{2\left(x^{2}+3\right)}{\left(x^{2}+9\right)\left(x^{2}+1\right)}\Delta \psi_{0}$$

where *ΔФ*_0_ *= kγI*_0_*L*_eff_ and *ΔΨ*_0_ *= βI*_0_*L*_eff_*/2* are the phase variations due to nonlinear refraction and nonlinear absorption, respectively, and *γ* is a nonlinear refractive index). By making the substitution *ρ = β/2kγ,* one can get the relation between *ΔФ*_0_ and *ΔΨ*_0_ (*ΔΨ*_0_ = *ρΔФ*_0_). In that case, Equation 6 can be replaced by^55^

(Equation 7) $$T=1+\frac{2\left(-\rho x^{2}+2x-3\rho \right)}{\left(x^{2}+9\right)\left(x^{2}+1\right)}\Delta \Phi_{o}$$

Notice that the Rayleigh length, and correspondingly the beam waist radius, can be determined from the CA curve by applying the relation for the distance between the valley and peak (ΔZ ≈ 1.7z_0_) in the case when the Kerr-related nonlinearity showed a prevailing influence over the other nonlinear optical processes.

The third-order nonlinear optical susceptibility *χ*^(3)^ was calculated using the relations for the real and imaginary parts using the relationships^54^

(Equation 8) $$Re\chi^{\left(3\right)}=2c{n_{0}}^{2}\varepsilon_{0}\gamma$$

(Equation 9) $$Im\chi^{\left(3\right)}=\frac{c^{2}{n_{0}}^{2}\varepsilon_{0}\beta }{\omega }$$

where *n*_0_ and *ε*_0_ are the linear refractive index and the vacuum permittivity, respectively, and *ω* is an angular frequency of the laser beam. The NLA and NLR coefficients were determined using the OA and CA data.

### Method details

#### Preparation of the substrates

The glass substrates were washed with acetone, ethanol, and deionized water for 30 min, respectively. After that, the cleaned substrate was dried and treated using UV light for 30 min, which allowed further removal of the residual organic matter from the surface, reducing the surface tension.

#### Fabrication of the CsPbX_3_ precursors and thin films

Cesium halide (CsX), lead halide (PbX_2_), (X = Cl, Br, I), and dimethyl sulfoxide (DMSO) were all purchased from Sigma-Aldrich. The existence of interstitial Pb atoms in a pure inorganic perovskite due to the low solubility of CsBr in organic solvents is more severe than that in organic-inorganic hybrid perovskites. However, reducing the concentration of precursors inevitably results in a decrease in the perovskite layer thickness and poor surface coverage of the perovskite film. We synthesized CsPbBr_3_ powders following the procedure reported by Stoumpos et al.^58^ The obtained CsPbBr_3_ powder of 0.55 M dissolved in DMSO to get the precursor solution. Similarly, we applied the same procedure for CsPbCl_3_ and CsPbI_3_ precursors.

The thin films of 200 nm thick CsPbX_3_ perovskites were formed on a glass substrate by spin-coating at a rotation speed of 1500 rpm. The samples were baked at 90°C for 10 min and then stored under the nitrogen atmosphere.

### Quantification and statistical analysis

Each OA and CA Z-scan measurement were measured by three to four times, the experimental data Z-scan data (symbols) shown in manuscript is the average of these measurements. Further, we have used the basic Z-scan relations to fit them theoretically to retrieve the NLO parameters for thin films and precursors.
